# Develop and validate a radiomics space-time model to predict the pathological complete response in patients undergoing neoadjuvant treatment of rectal cancer: an artificial intelligence model study based on machine learning

**DOI:** 10.1186/s12885-023-10855-w

**Published:** 2023-04-21

**Authors:** Jiaxuan Peng, Wei Wang, Hui Jin, Xue Qin, Jie Hou, Zhang Yang, Zhenyu Shu

**Affiliations:** 1 Jinzhou medical university, Jinzhou, Liaoning Province China; 2 Department of Radiology, The First Affiliated Hospital of Chongqing Medical and Pharmaceutical College, Chongqing, China; 3 Bengbu medical college, Bengbu, China; 4 Center for General Practice Medicine, Department of Radiology, Zhejiang Provincial People’s Hospital, People’s Hospital of Hangzhou Medical College, Hangzhou, Zhejiang China

**Keywords:** Radiomics, Rectal cancer, Pathological complete response, Machine learning, Neoadjuvant therapy

## Abstract

**Objective:**

In this study, we aimed to investigate the predictive efficacy of magnetic resonance imaging (MRI) radiomics features at different time points of neoadjuvant therapy for rectal cancer in patients with pathological complete response (pCR). Furthermore, we aimed to develop and validate a radiomics space–time model (RSTM) using machine learning for artificial intelligence interventions in predicting pCR in patients.

**Methods:**

Clinical and imaging data of 83 rectal cancer patients were retrospectively analyzed, and the patients were classified as pCR and non-pCR patients according to their postoperative pathological results. All patients received one MRI examination before and after neoadjuvant therapy to extract radiomics features, including pre-treatment, post-treatment, and delta features. Delta features were defined by the ratio of the difference between the pre- and the post-treatment features to the pre-treatment feature. After feature dimensionality reduction based on the above three feature types, the RSTM was constructed using machine learning methods, and its performance was evaluated using the area under the curve (AUC).

**Results:**

The AUC values of the individual basic models constructed by pre-treatment, post-treatment, and delta features were 0.771, 0.681, and 0.871, respectively. Their sensitivity values were 0.727, 0.864, and 0.909, respectively, and their specificity values were 0.803, 0.492, and 0.656, respectively. The AUC, sensitivity, and specificity values of the combined basic model constructed by combining pre-treatment, post-treatment, and delta features were 0.901, 0.909, and 0.803, respectively. The AUC, sensitivity, and specificity values of the RSTM constructed using the K-Nearest Neighbor (KNN) classifier on the basis of the combined basic model were 0.944, 0.871, and 0.983, respectively. The Delong test showed that the performance of RSTM was significantly different from that of pre-treatment, post-treatment, and delta models (*P* < 0.05) but not significantly different from the combined basic model of the three (*P* > 0.05).

**Conclusions:**

The RSTM constructed using the KNN classifier based on the combined features of before and after neoadjuvant therapy and delta features had the best predictive efficacy for pCR of neoadjuvant therapy. It may emerge as a new clinical tool to assist with individualized management of rectal cancer patients.

**Supplementary Information:**

The online version contains supplementary material available at 10.1186/s12885-023-10855-w.

## Introduction

For patients with locally advanced rectal cancer, the standard recommended treatment regimen is preoperative neoadjuvant chemoradiotherapy (nCRT) combined with total mesorectal excision [1]; however, in some patients, pathological complete response (pCR) can be achieved after only nCRT. Besides the advantage of not having to undergo surgery, such patients can also be followed up directly, which is a “wait-and-see” treatment strategy [2]. However, as some patients do not respond adequately to nCRT, some opportunities for good treatment are missed. Therefore, there is an urgent need for a reliable method to accurately predict the efficacy of nCRT.

For evaluating and predicting the efficacy of nCRT, various studies have used morphological features represented by TNM stage and tumor regression grade (TRG)[3], tumor marker features represented by carcinoembryonic antigen (CEA) and carbohydrate antigen-199 (CA199) levels [4, 5], and tumor microenvironment-related molecular features, such as EGFR, VEGF, and Ki67 [6, 7]. However, considering the spatial and temporal heterogeneity of tumor tissue, the use of the same category of indicators to predict the efficacy of the disease is associated with drawbacks of low sensitivity and specificity, thus preventing it from meeting the requirements of “precision medicine.”

At present, radiomics is an image analysis technology which involves the cross-integration of radiology, medicine, bioengineering, and other related disciplines. It has been widely used to evaluate the efficacy of neoadjuvant therapy for rectal cancer [8–10]. Previously, we have also reported on the use of T2-weighted imaging (T2WI)-based texture features for the prediction of pCR in patients [11]; however, low-throughput-based image features and small sample studies resulted in poor prediction sensitivity. Furthermore, this previous study neglected how the final prediction results would have been affected by the differences in time points of neoadjuvant therapy. Notably, the majority of similar studies in the literature are based on the features extracted from pre-neoadjuvant therapy for the prediction of pCR in patients [12, 13]. However, experience from studies wherein surgery was deferred suggests that pre-nCRT efficacy assessment underestimates the true incidence of pCR [14]. Therefore, it is necessary to comprehensively study the time points of feature extraction from a time- and cost-effectiveness perspective, which mainly includes pre-treatment, post-treatment, and the differences between them. Thus far, few studies have correlated the time point of extraction of radiomics features with the predicted outcomes. In addition, machine learning is attracting a lot of attention as a promising method to guide clinical decision-making. Machine learning enables better efficiency, accuracy, and reproducibility due to the large number of latent features that can be extracted to build classification or predictive models [15].

In conclusion, the primary purpose of this study was to determine the predictive performance of radiomics features extracted at different time points of nCRT of patients with pCR. Furthermore, we developed and validated a high-throughput machine learning-based radiomics space-time model (RSTM) to accurately predict pCR in rectal cancer patients.

## Materials and methods

### Patients

This retrospective study was approved by the Institutional Review Board of Zhejiang Provincial People’s Hospital (NO. 2021QT256), which waived the informed consent of all patients.

**Fig. 1:**
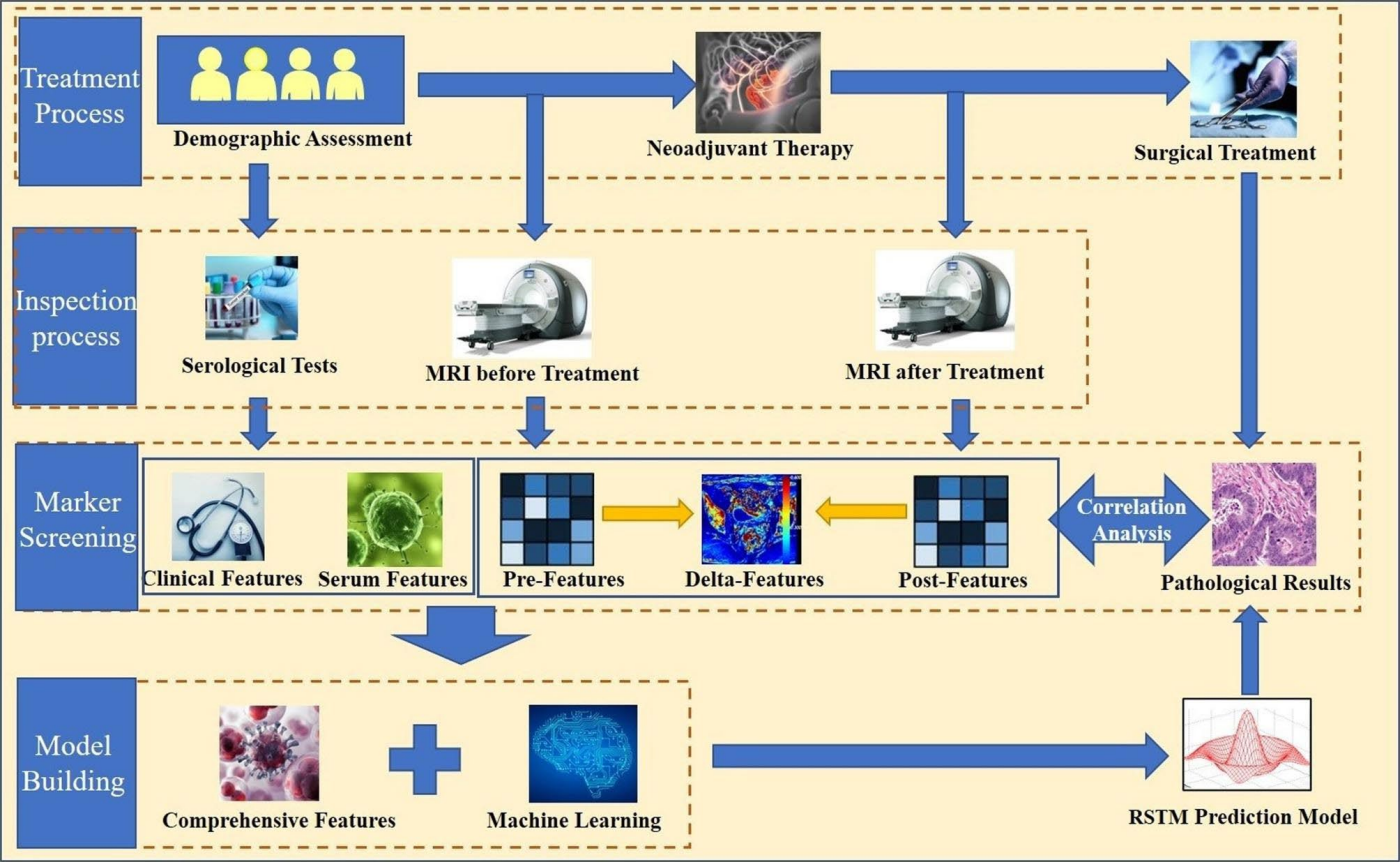
Study design diagram, including the model construction and validation process

This study is a retrospective analysis to develop a predictive model, and the specific process is shown in Fig. 1. To develop the RSTM, we retrospectively analyzed clinical and radiology imaging data of 2332 patients diagnosed as having rectal cancer between June 2017 and June 2022. The data were sourced from the picture archiving and communication system of Zhejiang Provincial People’s Hospital. The inclusion criteria were as follows: patients with locally advanced rectal cancer (cT3-4, N0, and M0) or (cT1-4, N1, and M0); 2) patients having undergone standard nCRT followed by total mesorectal excision; 3) patients with complete postoperative pathology results available; and 4) patients for whom complete MRI scans were performed before and after neoadjuvant therapy. The exclusion criteria were as follows: (1) failure to tolerate complete neoadjuvant therapy or treatment interruption; (2) history of other cancers or rectal cancer recurrence; (3) any contraindications to MRI scanning; and (4) lack of pathological results after rectal mesenteric resection. Finally, we recruited 83 patients which formed the dataset for the construction of the RSTM. Clinical variables were collected which included age, CEA, and CA199. The additional variables were obtained from the structured report of rectal cancer MRI, which includes the distance from the edge of the anus (DIS), circumferential resection margin (CRM) status, MRI-based extramural vascular invasion (mrEMVI) status, radiological tumor (T) stage and lymph node (N) stage. This study was conducted in accordance with the Declaration of Helsinki and was approved by the Ethics Committee. Owing to the retrospective design of the study, the need for informed consent was waived. The course of nCRT and pathological evaluation is shown in supplementary materials.

### Image preprocessing

All MRI images were acquired using a 3.0-T magnetic resonance imaging system (Skyra; Siemens Healthineers). The scans were acquired 2 weeks before and 4 weeks after nCRT, including T2-weighted imaging (T2WI), T1-weighted imaging (T1WI), diffusion-weighted imaging (DWI), and enhanced T1WI (T1 + C) sequences. To minimize the potential impact of scanning scheme parameters, we used the non-commercial AK software (GE Healthcare Analysis Kit) for preprocessing and aligning images for T2WI, T1WI, DWI, and T1 + C sequences before feature extraction. The image resolution was resampled to 1 × 1 × 1 mm^3^ by linear interpolation, and the image gray levels were discretized and normalized to 32 orders to perform image preprocessing. Next, all sequences were rigidly registered on T2WI perpendicular to the rectal axis, which was used as a template mask. This was done using the registration function of the AK software which ensured that the four sequences contained the same resolution, spacing, and origin. The normalized T2WI images were imported into the ITK-SNAP software (http://www.itksnap.org/), and the entire rectal tumor was segmented layer by layer to obtain the volume of interest (VOI). Finally, the VOI was imported into the AK software for feature extraction. Based on the alignment of sequences, we found that T1WI, DWI, and T1 + C could share the same VOI as T2WI to extract features. Detailed MRI scan parameters and schematics of registration are available in supplementary materials.

### Acquisition and selection of radiomics features

All feature extraction was based on the py-Radiomics Library (version 2.1.1) in PHIGO (Precision Health Institution, GE Healthcare) software, and specific feature information is shown in supplementary materials. We extracted 930 radiomics features from each MRI sequence and scanned four sequences per patient, thus amounting to 3720 radiomics features per patient for a single time point. Furthermore, in this study, two radiologists (Radiologist A and Radiologist B) independently and manually delineated tumor segmentation on pre- and post-treatment MRI images to ensure the stability and accuracy of radiomics features, and finally, feature sets A (from Radiologist A) and B (from Radiologist B) were obtained. The Spearman correlation test was used to calculate the correlation coefficient (CC) of each feature in sets A and B. The features with CC > 0.8 were selected as robust features for the construction of the RSTM model. In this study, we also calculated the delta feature, which is defined as the ratio of the difference between the pre- and the post-treatment features to the pre-treatment feature. The specific formula is as follows:

Delta = (Pre-treatment radiomic feature - Post-treatment radiomic feature)/Pre-treatment radiomic feature.

To obtain delta features, we only selected the radiomics features that existed both before and after treatment after feature stability screening to ensure that the three feature sets of pre-treatment, post-treatment, and delta features have the same number of features. Then, from the extracted features, the most important features related to the treatment response of nCRT were obtained. For feature selection, to reflect the largest difference between the tumor tissues before and after treatment, we first selected the features with the greatest difference among the three groups and then obtained the optimal features by dimensionality reduction based on the feature set of each group. Specific information on dimensionality reduction is provided in supplementary materials.

### Modeling strategy and model development

Based on the three optimal feature sets, we used logistic regression to construct a basic model for individual time points. We built a combined basic model at multiple time points, used a reverse stepwise selection method based on the stopping rule of the Akaike information criterion to select potential predictors, and evaluated the diagnostic performance at different time points based on the receiver operating characteristic curve (ROC) and Delong test. Next, we added some clinical features related to pCR to build a comprehensive RSTM based on the selected predictors. In this study, the RSTM was constructed with machine learning classifiers, including support vector machine (SVM), random forest (RF) classifier, and K-Nearest Neighbor (KNN) [16]. Furthermore, we used the bootstrap method for the RSTM construction and repeated it 1000 times to avoid reporting biased results and limit overfitting. In addition, for each time bootstrap validation, we also selected different hyperparameters for comparison, and finally, we selected the optimal classifier through the accuracy (ACC) and kappa value tests [17]. In this study, the output of RSTM was a binary prediction of pathological response to nCRT, defined as pCR or non-pCR, and the machine learning and bootstrap method part is elaborated in the supplementary materials.

### Model validation

We used the AUC values of the ROC curve to evaluate and compare the diagnostic performance of the RSTM with that of each basic model constructed at a single time point and jointly at three time points; we used the Delong test to evaluate the differences in performance. In addition, to assess the clinical applicability of the RSTM, we used the best cut-off value corresponding to the Youden index of the ROC curve as the threshold, and the pCR prediction score of each case calculated by the RSTM was used to divide all cases into high- and low-probability groups of pCR. Then, we used the final postoperative pathological report as the gold standard to evaluate the classification performance of the RSTM model.

### Statistical analysis

Statistical analysis was performed using SPSS software (version 24.0), MedCalc software (version 11.2), and Python (version 3.5). Continuous variables were compared using the two-sample t-test or Mann–Whitney U test, and categorical variables were compared using the chi-square test. All statistics were two-sided, and statistical significance was set at *P* < 0.05. For selecting radiomics features, we first used one-way analysis of variance (ANOVA) to select features with differences among the three sets of features and then performed pairwise comparisons between groups for features with statistical differences. We applied Bonferroni correction accordingly; *P* value of < 0.017 (0.05/3) was considered statistically significant for comparisons among all three groups. The kappa value in machine learning is defined as kappa = (observed accuracy – expected accuracy)/(1 - expected accuracy), and this value typically falls between 0 and 1, with a value of 0–0.2 indicating slight consistency (slight), 0.21–0.4 indicating fair consistency, 0.41–0.6 indicating moderate consistency, 0.61–0.8 indicating substantial consistency, and 0.81–1 indicating almost perfect consistency.

## Results

### Comparison of baseline clinical characteristics

A total of 83 cases were included in this study. There was no statistically significant difference between pCR and non-pCR patients in terms of their demographic characteristics and conventional radiology characteristics (*P* > 0.05); however, the DIS differed significantly (*P* < 0.05). Detailed results in this regard are provided in Table 1.

**Table 1:** Baseline clinical characteristics of the study population

Characteristics	Study dataset (n = 83)			
	ALL patients	pCR (n = 22)	No-pCR (n = 61)	*P*
Age (years, SD)	63.83 (13.64)	61.76 (9.21)	64.54 (13.64)	0.21
Sex (N, %)				
Male	60 (72.3)	15 (81.8)	45 (73.8)	0.208
Female	23 (27.7)	7 (18.2)	16 (26.2)	
CEA (N, %)				
Abnormal	48 (57.8)	15 (68.2)	33 (54.1)	0.251
Normal	35 (42.2)	7 (31.8)	28 (45.9)	
CA199 (N, %)				
Abnormal	22 (26.5)	9 (36.4)	13 (21.3)	0.235
Normal	59 (73.5)	13 (59.1)	46 (75.4)	
DIS (cm, SD)	5.02 (2.28)	6.03 (1.92)	4.62 (2.28)	0.012*
CRM status (N, %)				
Positive	50 (60.2)	12 (54.5)	38 (62.3)	0.524
Negative	33 (39.8)	10 (45.5)	23 (37.7)	
mrEMVI status (N, %)				
Positive	36 (22.2)	8 (36.4)	28 (45.9)	0.474
Negative	47 (77.8)	14 (63.6)	33 (54.1)	
Tumor stage (N, %)				
T_1 − 2_	9 (10.8)	2 (90.9)	7 (11.5)	0.736
T_3 − 4_	74 (89.2)	20 (90.9)	54 (88.5)	
Lymph node (N, %)				
N_0_	11 (13.3)	3 (13.6)	8 (13.1)	0.289
N_1 − 2_	72 (86.7)	19 (86.4)	53 (86.9)	

Note: CEA, carcinoembryonic antigen; CA199, carbohydrate antigen 199; DIS, the distance from the end of the convex edge of the tumor to the edge of the anus; CRM, circumferential resection margin; mrEMVI, MRI-based extramural vascular invasion. Data are presented as counts or means (standard deviations in parentheses)

### Feature selection

**Fig. 2:**
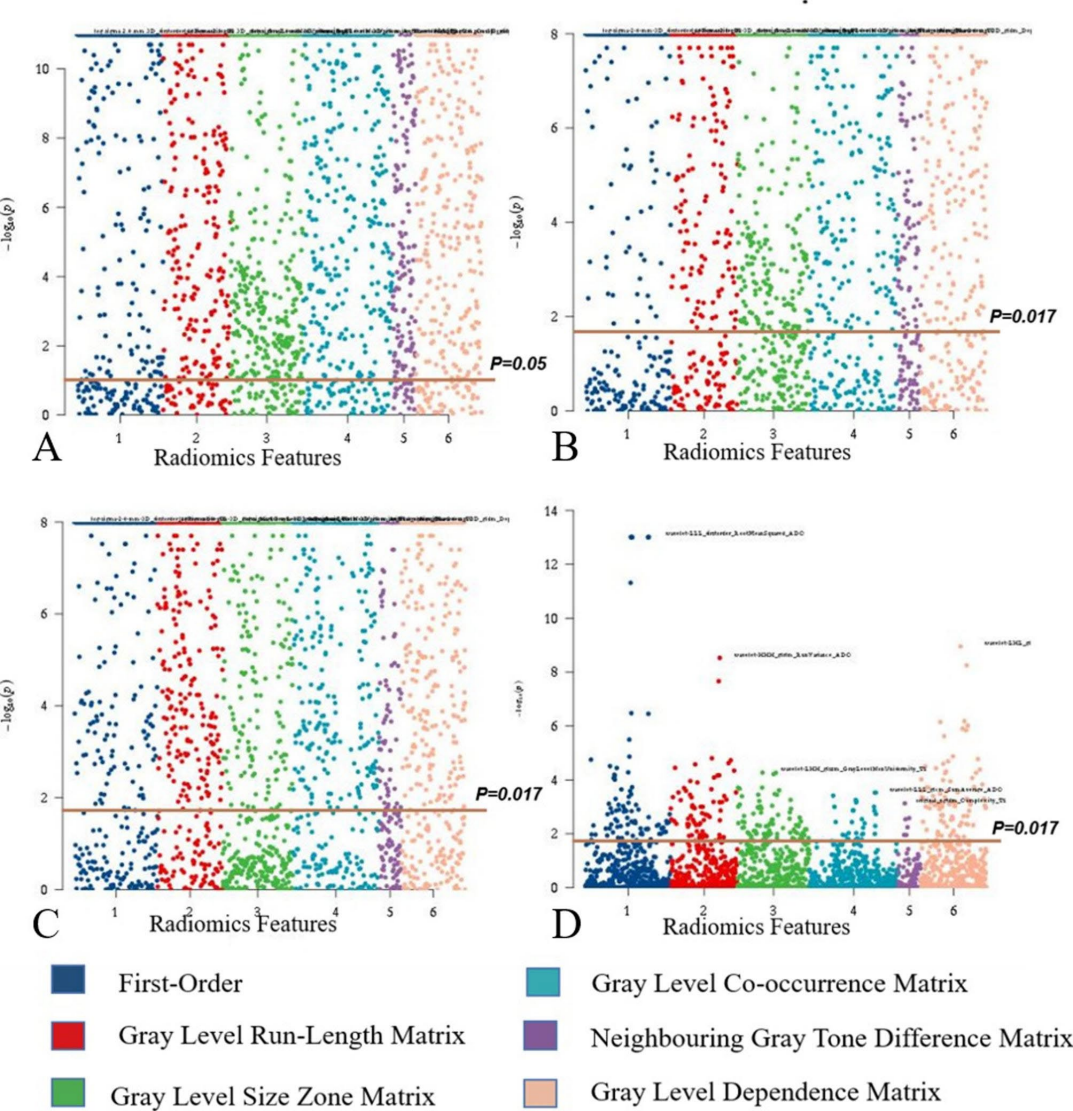
Feature screening process in Manhattan plots. Figure A represents the results of a one-way ANOVA among the three groups. Figure B shows the features that differed between the pre-treatment features and delta features. Figure C shows the features that differed between the post-treatment features and delta features. Figure D shows features differed between pre- and post-treatment features

**Fig. 3:**
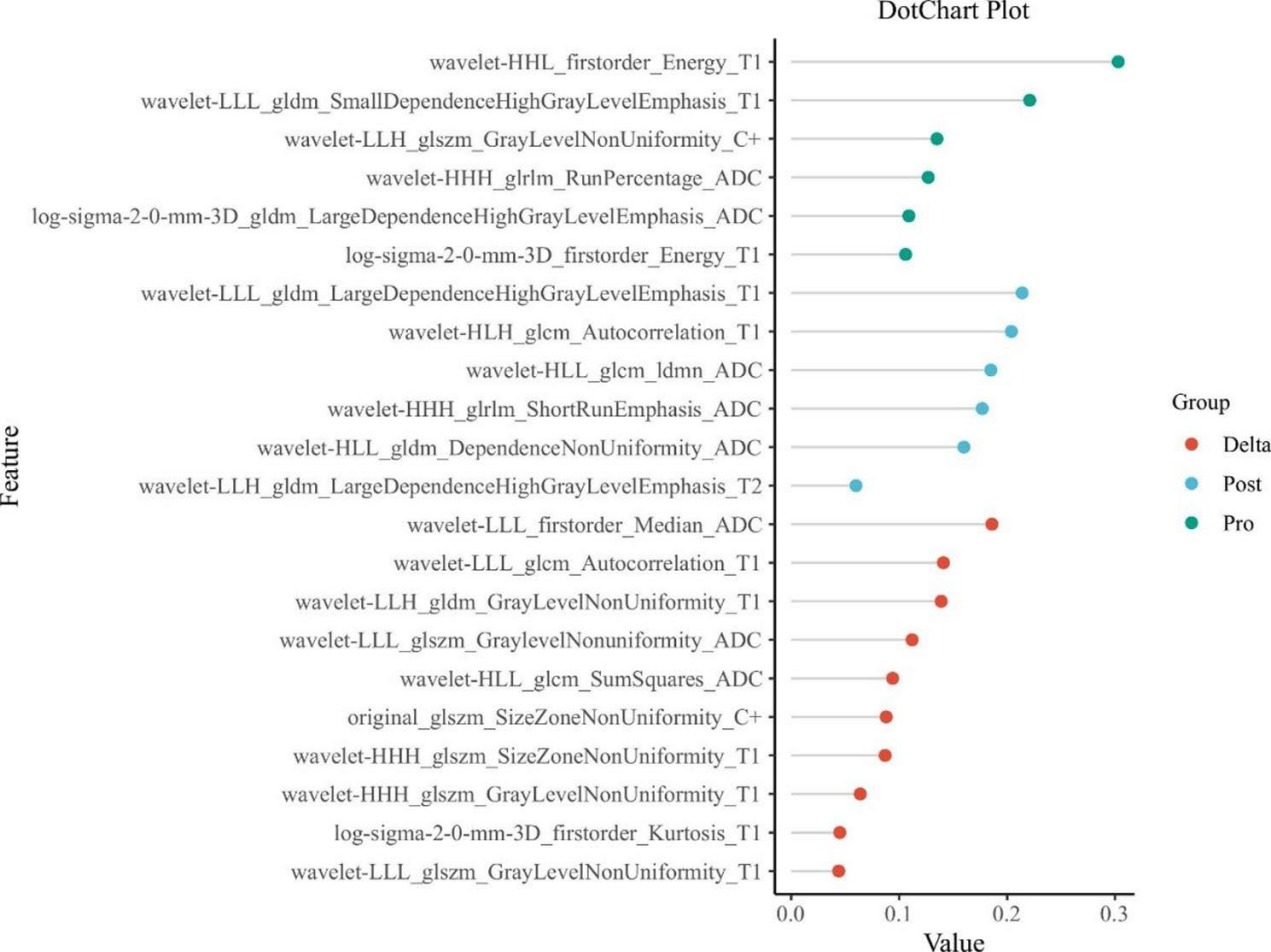
The dot chart shows the specific features remaining in the pre-treatment, post-treatment, and delta feature sets after dimensionality reduction by GBDT, where the abscissa represents the weight set for each feature set

From among 3720 features, a total of 2960 robust features were screened, and then, 2396 features were selected using one-way ANOVA; next, after pairwise comparisons of these features, 333 features were selected on the basis of the corrected *P* value. Figure 2 shows the 333 features selected for subsequent dimension reduction processing, and after gradient boosting decision tree (GBDT) dimensionality reduction, 6 features were obtained in the pre-treatment group, 6 features in the post-treatment group, and 10 features in the delta group. These 22 features are shown in Fig. 3. Thereafter, 8 features (Table S2) were screened by multivariate logistic regression analysis to construct the combined basic model, including two pre-treatment features and six delta features.

### Basic model construction and comparison

The AUC values of the pre-treatment, post-treatment, delta, and their combined basic models constructed based on the three optimal feature sets were 0.771, 0.681, 0.871, and 0.907, respectively. Their sensitivity values were 0.727, 0.864, 0.909, and 0.909, respectively, and their specificity values were 0.803, 0.492, 0.656, and 0.803, respectively; detailed results are provided in Table 2. The Delong test revealed a statistically significant difference in the diagnostic performance between the combined basic model and the pre- and post-treatment basic models (*P* < 0.05). In addition, there was also a statistical difference in the diagnostic performance between the delta basic model and the post-treatment basic model (*P* < 0.05).

**Table 2:** Results of the ROC curve analysis for each basic model

ROC curve analysis	Classification of the basic model			
	**Pre-treatment model**	**Post-treatment model**	**Delta model**	**Combined basic model**
AUC	0.771 (0.666, 0.856)	0.681 (0.578, 0.775)	0.871 (0.78, 0.935)	0.907 (0.848, 0.956)
Sensitivity	0.727	0.864	0.909	0.909
Specificity	0.803	0.492	0.656	0.803
F1_score	0.612	0.528	0.613	0.667
Recall	0.682	0.864	0.864	0.909
Accuracy	0.771	0.59	0.711	0.759

Note: ROC, receiver operating characteristic; AUC, area under the curve

### The RSTM construction and evaluation

**Fig. 4:**
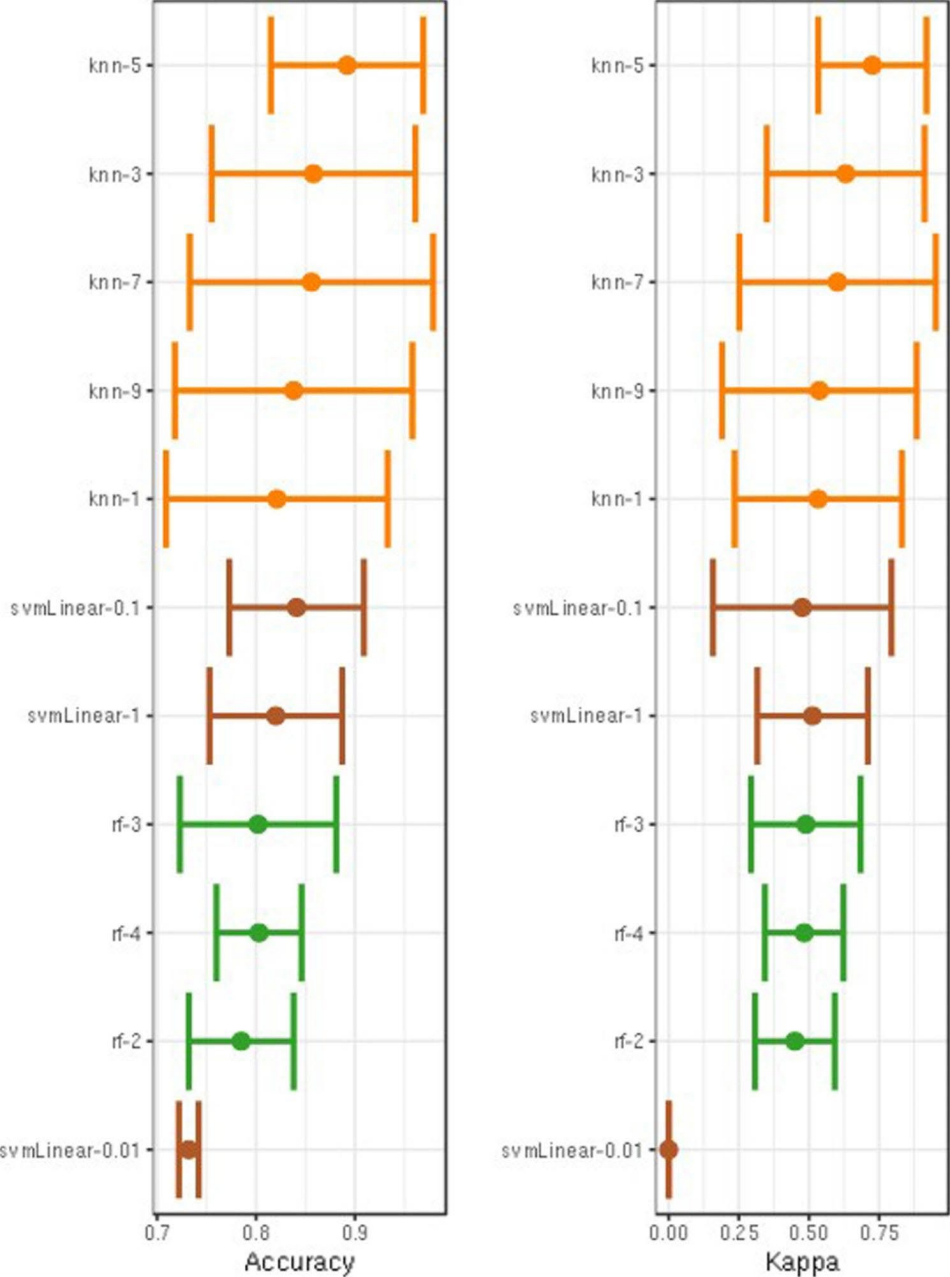
ACC and Kappa result in different hyperparameter settings in different machine learning methods, among which KNN-5 has the highest accuracy and Kappa value

**Fig. 5:**
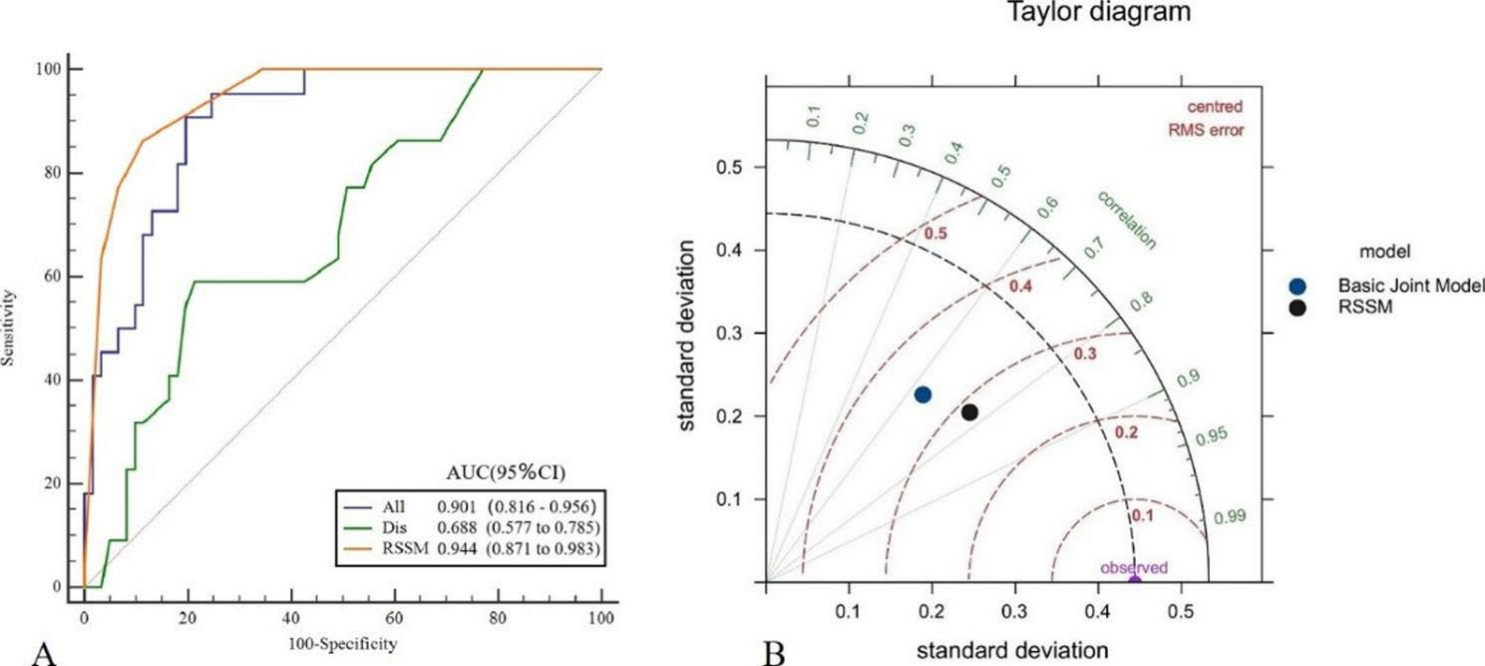
Figure A shows the ROC curve comparison of RSTM and the combined basic model and DIS. Figure B shows the Taylor plot of model comparison, showing that the performance of the RSTM is the closest to the observation point, reflecting the superiority of KNN modeling, where abscissa is the standard deviation, the radiation line is the correlation coefficient, and the dotted line is the root mean square error

Based on multivariate logistic regression analysis, the DIS and combined basic model scores were selected as independent predictors to construct the RSTM; detailed results are provided in Table 3. The machine learning results show that KNN has higher accuracy than SVM and RF. Figure 4 and Table S3 show the accuracy evaluation of different machine learning and hyperparameters. When the hyperparameter is selected as 5, the KNN classifier has the highest ACC and kappa values of 0.892 and 0.726, respectively. The AUC, sensitivity, and specificity values of the RSTM constructed based on KNN-5 were 0.944, 0.864, and 0.885, respectively, which are shown in Fig. 5A. The Delong test revealed no statistically significant difference between the RSTM and the combined basic model (*P* > 0.05). To further showcase the superiority of KNN, we used Taylor plots to visualize the performance of the model, which revealed that the RSTM had better performance than the combined basic model. The Taylor plot is shown in Fig. 5B. According to the Youden index of the RSTM model, all patients were identified as high-probability pCR patients when their prediction score was > 0.2. Based on the pathological gold standard, there was a significant difference in the actual number of pCR patients between the high-probability pCR group and the low-probability pCR group (*P* < 0.05), indicating that the RSTM has good clinical applicability. The clinical classification performance of the RSTM is shown in Table 4.

**Table 3:** Results of univariate and multivariate logistic regression analyses

Variables	Univariate logistic regression			Multivariate logistic regression	
	OR (95%CI)	*P* value		OR (95%CI)	*P* value
Age	0.984 (0.948, 1.023)	0.597		NA	NA
Sex	1.113 (0.609, 2.034)	0.728		NA	NA
CEA	1 (0.99, 1.009)	0.919		NA	NA
CA199	1.002 (0.999, 1.004)	0.221		NA	NA
DIS	1.335 (1.055, 1.689)	0.016*		1.397 (1.032, 1.891)	0.03*
CRM status	0.564 (0.211, 1.512)	0.255		NA	NA
mrEMVI status	0.673 (0.247, 1.838)	0.440		NA	NA
T stage	1.296 (0.248, 6.771)	0.758		NA	NA
N stage	0.956 (0.230, 3.981)	0.951		NA	NA
Combined basic model score	25.861 (6.561, 101.932)	< 0.001		28.554 (6.618, 123.204)	< 0.001*

Note: CEA, carcinoembryonic antigen; CA199, carbohydrate antigen 199; DIS, the distance from the end of the convex edge of the tumor to the edge of the anus; CRM, circumferential resection margin; mrEMVI, MRI-based extramural vascular invasion

**Table 4:** Clinical classification performance of RSTM model

	Group	Number of cases (n, %)		Pathological results			*P*
				pCR (n, %)		Non-pCR (n, %)	
Predict results of RSTM	Non-pCR		57 (68.68)		3 (5.26)	54 (94.74)	< 0.001*
	pCR		26 (31.32)		19 (73.08)	7 (26.92)	
Number of cases (n, %)			83 (100)		22 (26.5)	61 (73.5)	

Note: pCR, pathological complete response; RSTM, radiomics space–time model

## Discussion

The results of this study confirm our hypothesis that consolidated information obtained from multiple points in time can more comprehensively evaluate patients with pCR than information obtained at a single point in time. The AUC value of the post-treatment model was lower than that of the pre-treatment model, which may be attributed to the tumor having necrosis and neovascularization after the treatment, which may have resulted in an inaccurate reflection of tumor heterogeneity [18]. In addition, machine learning was found to significantly improve the efficiency of the combined basic model, suggesting that it can offer significant advantages in tumor treatment and prognosis in clinical practice.

Notably, pCR is associated with local disease control and long-term survival [19]. At present, the determination of pCR depends heavily on the specimen after surgical resection, and there is no reliable and accurate method to predict it before nCRT. Compared with traditional radiology technology, radiomics can extract high-dimensional features that are imperceptible to the human eye. At present, some prediction models based on the radiological features of MRI are aimed at predicting the pCR status of rectal cancer patients after nCRT [20–22]; however, As mentioned in the introduction, the efficacy evaluation before nCRT underestimates the true incidence of pCR, while most of these studies only focus on the information of MRI before treatment. Therefore, it is controversial whether focusing on a certain period rather than a certain moment will have a greater impact on the predictive effect of pCR. Considering the above reasons, the current study added post-treatment MRI information and delta information generated by the difference between the pre- and post-nCR, and also modeled these three temporal information separately. The final results showed that the model constructed with delta information had better efficiency compared to the other two time point information, which suggests that one time period contains more biological information than one time point and can better predict pCR. In addition, the RSTM also contains delta features, which further illustrate the importance of delta features.

In a previous study, T2-weighted MRI radiomics features were considered as potential imaging biomarkers for the early prediction of rectal cancer non-response to nCRT [23]. Compared with standard MRI alone, standard MRI + DWI shows better diagnostic performance in predicting pCR after neoadjuvant radiotherapy and chemotherapy [24]. Compared with these models, RSTM has several distinct advantages. First, both standard MRI alone and standard MRI + DWI extract radiomics features from a single treatment time point and do not analyze differences in radiomics features before and after nCRT. Conversely, RSTM involves a longitudinal analysis of changes in MRI features during radiotherapy, which covers the entire course of treatment and therefore better reflects the development trends of heterogeneity with neoadjuvant therapy for rectal cancer. Second, some studies have reported suboptimal results using morphological features of T2WI sequences in assessing pCR [25]. Wan et al. reported that the radiomics features from combined T2-W, ADC, and cT1-W sequences showed better prediction performance than a single sequence [26]. Therefore, we used multisequence feature extraction to build a prediction model and used rigid registration to ensure the consistency of ROI between different sequences. This study also shows that the RSTM includes two pre-treatment features and six delta features, among which the delta features contribute a lot to the construction of the RSTM model. We can see that the features at a single time point still cannot reflect tumor heterogeneity and changes in tumor characteristics, and the delta feature has great potential in this regard. Due to the small relative variability, it can be directly measured to reflect the longitudinal changes in images over multiple periods, and it has recently been effectively applied to various cancer treatments [27–30]. Chang et al. investigated a delta radiomics-based machine learning model for predicting overall survival in patients with recurrent malignant glioma [31]. In their work, compared with the model based on characteristics of a single time point, the delta radiomics-based model showed higher performance, which is also consistent with our research results. However, it should be noted that the RSTM does not include post-treatment features, which further suggests that the information on post-treatment features is not sufficient to effectively predict pCR, which is further corroborated by the lowest diagnostic performance of the post-treatment model. Furthermore, it is known that incorporating more variables, such as clinical characteristics, can increase the stability of the model. Unfortunately, there was a statistically significant difference between pCR and non-pCR only in terms of DIS. This finding may be attributed to the higher correlation between radiomics and clinical baseline information, which is consistent with the smaller contribution of clinical information to the model in previous studies [32].

Currently, most similar studies are based on the logistic regression model. In this study, different machine learning methods are used to improve the model’s performance. In a study by Mao et al., radiology analysis was carried out on CT images before routine clinical nursing treatment, and a radiology nomogram was developed to predict the response of locally advanced rectal cancer (LARC) patients to nCRT; they reported an AUC value of 0.87 [33]. Wang et al. studied the delta radiomics of MRI to evaluate pCR after nCRT in LARC patients and reported an AUC value of 0.91 [34], indicating inferior diagnostic performance to the RSTM (AUC = 0.944), which could be attributed to them ignoring the impact of modeling methods on prediction results. Notably, predictive models are an important part of radiomics. Building highly accurate and reliable models can aid decision-making in clinical practice, and machine learning may help in this regard. We compared multiple machine learning classifiers, among which the RSTM based on KNN was found to have higher accuracy than the model based on logistic regression. This further confirms that adding machine learning methods to build radiomics models can improve the prediction accuracy of pCR.

Notwithstanding, our study has some limitations. First, the study sample size is small, which may have led to overfitting of the model during training. However, we have performed the bootstrap method, which further reduces the bias of the results. Second, the study lacks external validation, and our predictive model needs to incorporate more cases to improve its reproducibility and generalizability. Finally, due to the edema and fibrosis that accompanies nCRT, some tumors show blurred margins, which may affect imaging segmentation. However, MRI shows higher tissue contrast than other imaging modalities (e.g., computed tomography), thus allowing for more accurate detection of tumor margins.

The currently constructed RSTM achieves a more multidimensional and robust prediction of pCR from the perspective of full neoadjuvant radiotherapy, which will provide clinicians with reliable information to assess pCR and help advance individualized treatment of rectal cancer.

## Electronic supplementary material

Below is the link to the electronic supplementary material.

Supplementary Material 1

## Data Availability

The datasets used and analyzed in this article is available from the corresponding author on reasonable request.
