# Supplementary material for: Develop and validate a radiomics space-time model to predict the pathological complete response in patients undergoing neoadjuvant treatment of rectal cancer: an artificial intelligence model study based on machine learning
Source: BMC Cancer. 2023 Apr 21;23:365. doi: 10.1186/s12885-023-10855-w (PMC10120125; doi:10.1186/s12885-023-10855-w)
Supplement: Supplementary file 1 — Supplementary Material 1 [file 12885_2023_10855_MOESM1_ESM.docx]

**Supplementary material**

**1. Neoadjuvant concurrent chemoradiotherapy**

All patients received long-term treatment with neoadjuvant concurrent chemotherapy for rectal cancer. The nCRT comprised radiotherapy at a dose of 2.0 Gy/day given 5 days per week for 5 weeks, with the total dose being 50 Gy, which was delivered in one posterior and two lateral positions. Chemotherapy was administered simultaneously with the radiotherapy and comprised intravenous oxaliplatin at 50 mg/m^2^ per week and oral capecitabine at 825 mg/m^2^ per day given 5 days per week for 5 weeks. All enrolled patients successfully completed the planned nCRT.

**2.Postoperative pathological evaluation**

Based on surgical resection specimens, pathologists (with more than 5 years of experience in gastrointestinal pathology) abstracted, assessed, and prepared pathology reports. The specimens were scored according to the tumor regression grading after chemoradiotherapy as proposed by Dworak et al. TRG4 indicated complete regression, and no viable tumor cells were observed in the specimen; only fibrous tissue degeneration was observed. TRG3 indicated significant regression, with >50% of tumor cells showing degeneration and evident tumor tissue fibrosis. TRG2 indicated moderate regression, and 26%–50% of the tumor tissue was observed to have evident fibrosis. TRG1 indicated basically no morphological changes in the tumor tissue, and <25% of the tumor tissue showed evident fibrosis. Finally, TRGO indicated no regression. In this study, TRG4 indicated pathologic complete response (pCR), and the rest were considered as indicative of no response.

**3.** **MRI image acquisition**

All patients underwent placental MRI in the supine position using 3.0T MR imaging systems (Skyra, Siemens Healthineers, Germany) equipped with an 8-channel phased-array coil. The MRI sequences included sagittal T2WI, axial T2 blade TSE, axial resolve DWI, and enhanced T1WI. All patients were intravenously injected with a contrast agent (gadolinium, Magnevist, Bayer, Germany). The detailed parameters for each sequence are illustrated in Table S1.

**Table S1. MRI parameters of each sequence**

| **Scanner** | **Sequence** | **TR (ms)** | **TE (ms)** | **Slice Thickness (mm)** | **Slice Gap (mm)** | **Fov (mm)** | **Slices** | Matrix |
| --- | --- | --- | --- | --- | --- | --- | --- | --- |
| SIEMENS 3.0 T  (Skyra) | Sagittal T2 WI | 6060 | 90 | 3 | 0.6 | 180´180 | 25 | 320´224 |
|  | Axial T2_blade_TSE | 4790 | 134 | 3 | 0.6 | 200´200 | 35 | 384´451 |
|  | Axial T1 WI | 662 | 9.6 | 3 | 0.6 | 180´180 | 35 | 320´224 |
|  | Axial resolve DWI (DWI b = 600) | 7330 | 56 | 3 | 0.8 | 200´200 | 35 | 112´100 |
|  | Enhanced T1 WI | 616 | 9.6 | 3 | 0.6 | 180´180 | 35 | 320´224 |

**4. Image segmentation program**

First, the AK software (GE Healthcare Analysis Kit, Shanghai, China) was used to rigorously register the images of T2WI, T1WI, ADC, and T1CE sequences to reduce the potential influence of the parameters of a scanning scheme. Then, the standardized T2WI images were imported into the ITK software (http://www.itksnap.org/) to manually segment the entire rectal tumor layer by layer and determine the volume of interest (VOI). Since the four sequences were rigorously registered, tumor VOI obtained from T2WI could be applied directly to other sequences. All cases undertook the same VOI segmentation method.


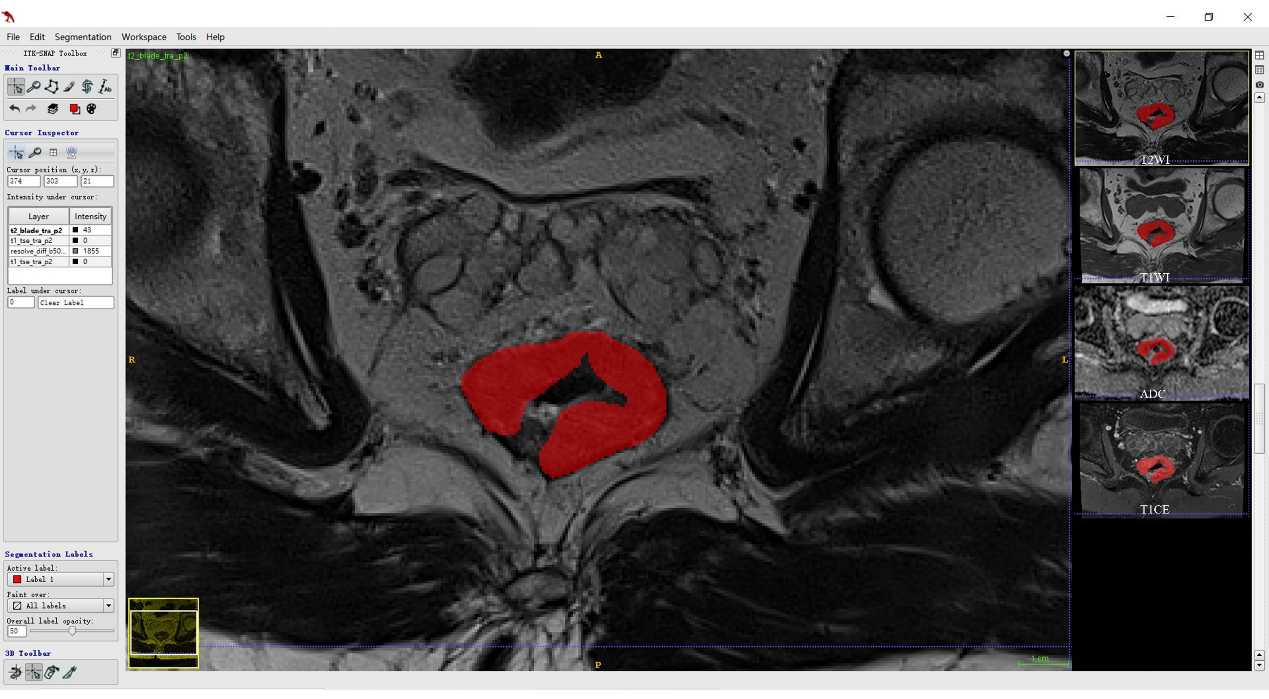


**Figure S1.** Representative manual segmentation of the entire rectal tumor in the T2WI, T1WI, ADC, and T1CE using the ITK software.

**5. Standardization of data**

The extracted features were standardized before dimension reduction, which removed the unit limits of the data of each feature and converted the value of a feature into a dimensionless pure value. This allowed the indices of different units or orders to be compared and weighted. We used Z-score normalization to make the feature intensities fit a standard normal distribution with μ and σ, wherein μ is the mean value of the features and σ is the standard deviation. The normalized values (also called Z-scores) of the feature intensities (x) were calculated as follows:


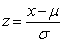


**6. Details of dimension reduction**

First, we performed analysis of variance on the extracted features. The variance value was the average of the square of the difference between the value of each variable and the mean. It is the most important method for measuring the dispersion of numeric data. The larger was the variance, the greater was data fluctuation and vice versa. Therefore, it is necessary to preferentially eliminate features with a variance of ≤0. In this study, the variance of each feature was calculated, and then, the features greater than the threshold 1 were retained.

Second, the minimum redundancy maximum relevance (mRMR) algorithm was used to extract robust features from the training set. The aim of the maximum relevance procedure was to select features having the maximum correlation with the actual PD diagnosis. At the same time, the minimum redundancy process ensured that the selected features had minimal redundancy among the other features, and we selected features with correlation coefficients greater than 0.1 and 0.8 as high correlation and low redundancy features, respectively. Then, the mRMR method was used to obtain an optimal feature set with a high correlation and low redundancy.

Finally, the gradient boosting decision tree (GBDT) algorithm was used to reduce the dimension of the remaining features. GBDT is an algorithm that classifies or regresses data by a linear combination of basis functions and reduces the residual generated in the training process. Finally, 6 features were obtained in the pre-treatment group, 6 features in the post-treatment group, and 10 features in the delta group. The details of dimension reduction are shown in Figure S2–S4.


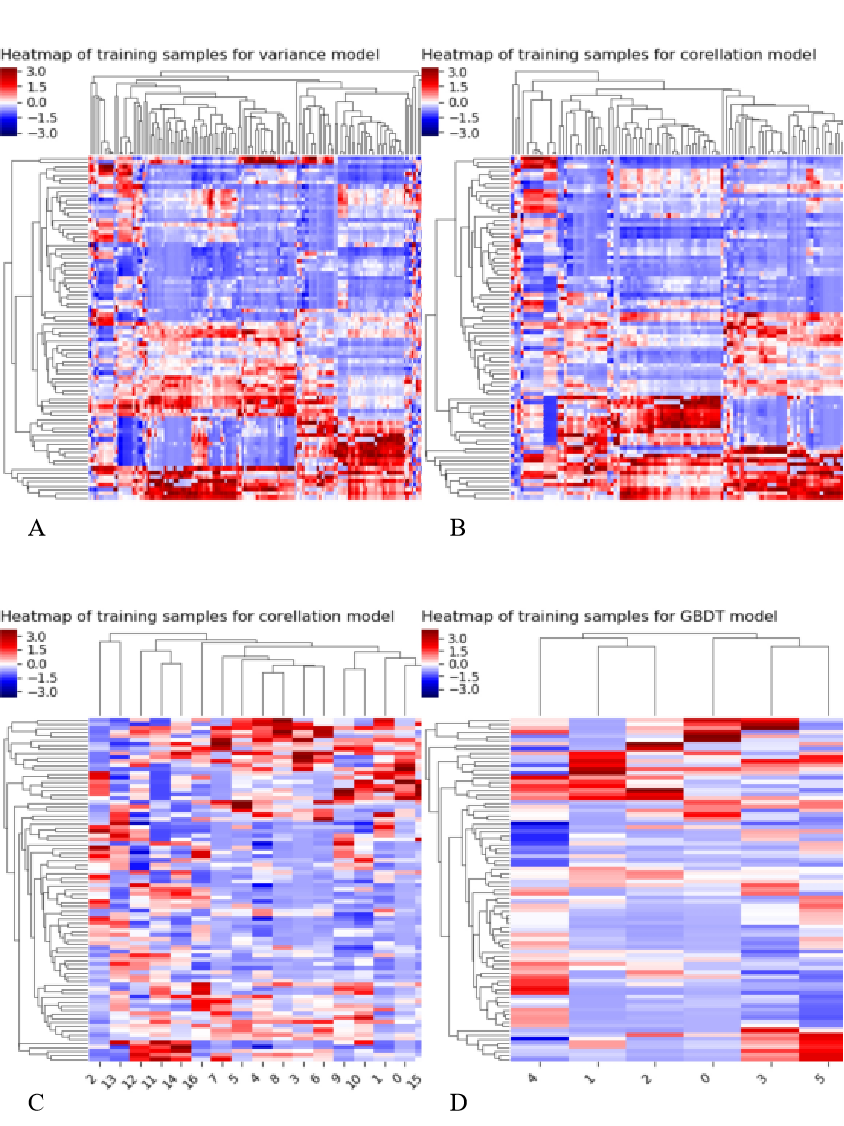


Figure **S2.** Process of heatmap for dimension reduction in the pre-treatment model. A. Features selected based on analysis of variance. B. Features extracted from the correlation analysis with clinical outcomes. C Features extracted from the correlation analysis between features. D. The remaining features after dimensionality reduction using GBDT. Abscissa represents feature ordering, ordinate represents case sequencing, and color represents feature value size.


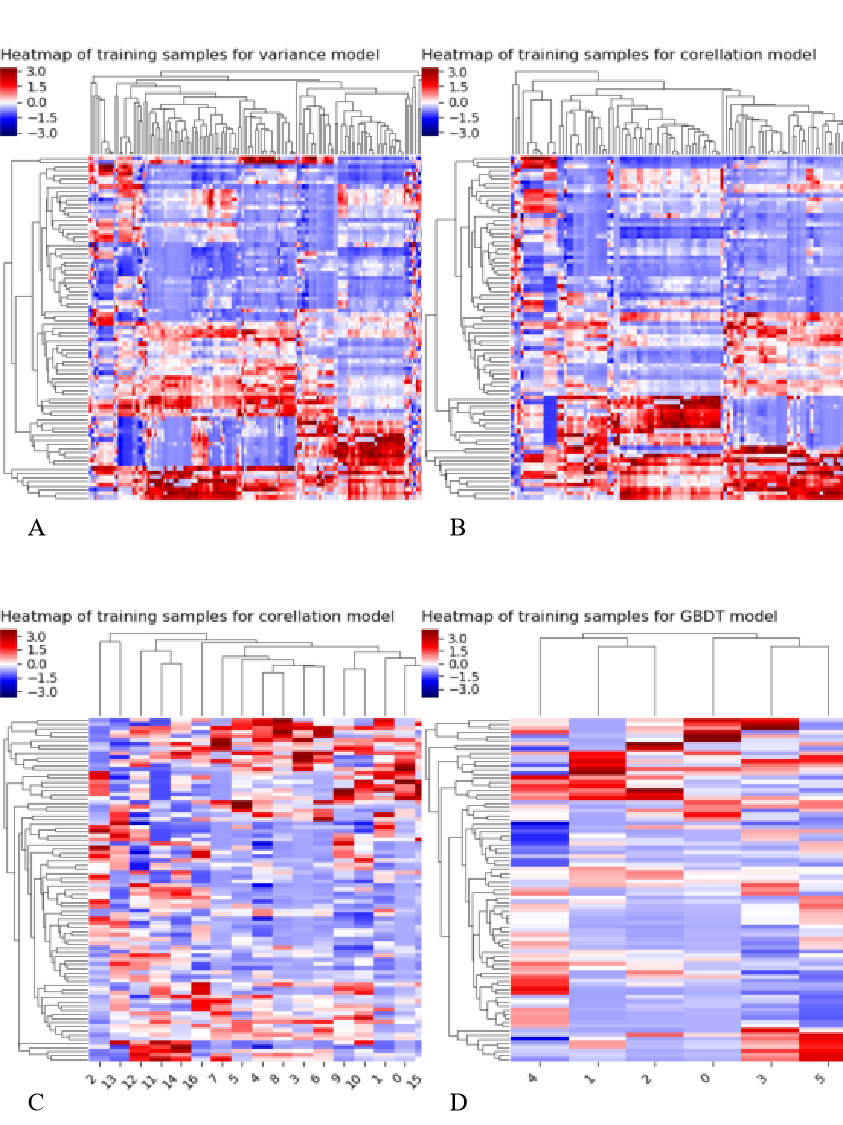


Figure **S3.** Process of heatmap for dimension reduction in the post-treatment model. A. Features selected based on analysis of variance. B. Features extracted from the correlation analysis with clinical outcomes. C. Features extracted from the correlation analysis between features. D. The remaining features after dimensionality reduction using GBDT. Abscissa represents feature ordering, ordinate represents case sequencing, and color represents feature value size.


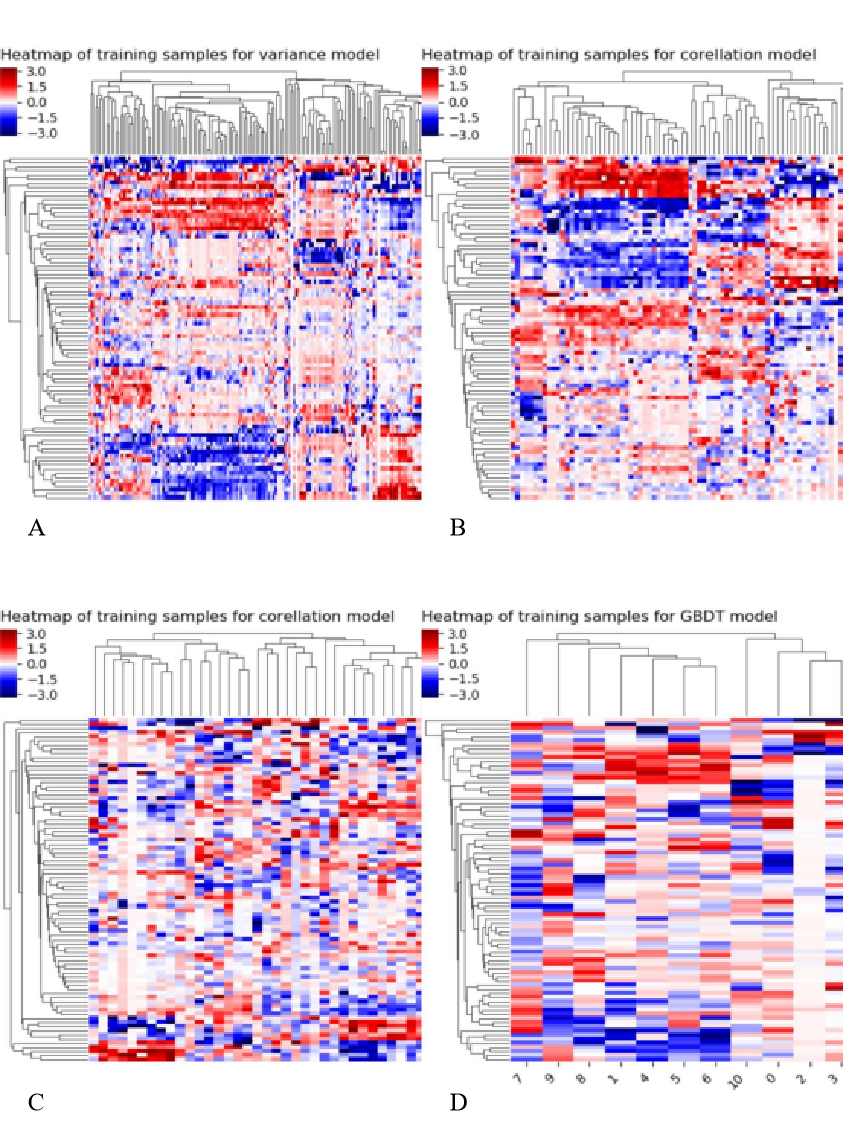


Figure **S4.** Process of heatmap for dimension reduction in the delta model. A. Features selected based on analysis of variance. B. Features extracted from the correlation analysis with clinical outcomes. C. Features extracted from the correlation analysis between features. D. The remaining features after dimensionality reduction using GBDT. Abscissa represents feature ordering, ordinate represents case sequencing, and color represents feature value size.

**Table S2. Features selected based on stepwise logistic regression for constructing combined basic model**

| **Time point** | **Variables** | **Multivariate logistic regression** | |
| --- | --- | --- | --- |
|  |  | **OR（****95%CI）** | ***P* value** |
| pre-treatment | wavelet-HHL_firstorder_Energy_T1 | 2.236 (1.279, 3.909) | 0.005 |
|  | wavelet-LLH_glszm_GrayLevelNonUniformity_C+ | 0.489 (0.249, 0.958) | 0.037 |
| Delta  治 | wavelet-LLL_firstorder_Median_ADC | 0.489 (0.247, 0.97) | 0.041 |
|  | wavelet-HHH_glszm_GrayLevelNonUniformity_T1 | 2.664 (1.211, 5.862) | 0.015 |
|  | wavelet-HLL_glcm_SumSquares_ADC | 0.262 (0.106, 0.645) | 0.004 |
|  | wavelet-LLL_glcm_Autocorrelation_T1 | 02.153 (1.061, 4.367) | 00.034 |
|  | wavelet-LLL_glszm_GrayLevelNonUniformity_ADC | 10.323 (0.142, 0.734) | 0.007 |
|  | original_glszm_SizeZoneNonUniformity_C+ | 00.489 (0.25, 0.955) | 00.036 |

**7. Machine learning**

Bootstrap method involved stratifying and dividing the data into 2 folds of equal size: 1 folds (50%) for training, and 1 fold (50%) for testing. Stratification was used to ensure a similar distribution of events across the 2 folds. This process was repeated for 1000 iterations, always using a different data fold for model training and testing. The machine learning scores were concatenated from all 1000 testing data folds to allow for the assessment of model performance over the entire dataset. Each of our three ML models was cross-validated separately using the same set of iterations and same folds of patient data. For each classification method, we trained the model on a subsampled training cohort (size N/2) from the dataset and evaluated the performance on the remaining data using the accuracy and kappa values.

**Table S3. Accuracy evaluation of different machine learning and hyperparameters**

| Machine Learning | Hyperparameter | Accuracy (SD) | Kappa (SD) |
| --- | --- | --- | --- |
| KNN | knn-1 | 0.821 (0.112) | 0.533 (0.298) |
|  | knn-3 | 0.858 (0.103) | 0.631 (0.281) |
|  | knn-5 | 0.892 (0.077) | 0.726 (0.193) |
|  | knn-7 | 0.856 (0.123) | 0.602 (0.35) |
|  | knn-9 | 0.838 (0.12) | 0.537 (0.347) |
| Random Forest | rf-2 | 0.785 (0.053) | 0.45 (0.142) |
|  | rf-3 | 0.802 (0.079) | 0.489 (0.195) |
|  | rf-4 | 0.803 (0.043) | 0.483 (0.14) |
| SVM | svm-0.01 | 0.732 (0.01) | 0.001 (0.001) |
|  | svm-0.1 | 0.841 (0.068) | 0.476 (0.318) |
|  | svm-1 | 0.82 (0.067) | 0.513 (0.197) |

*Abbreviations*: AUC: area under the curve; SD: standard deviation

**Table S4. The information of radiomics features**

| Feature Groups (N) | Feature names | Feature Groups (N) | Feature names |
| --- | --- | --- | --- |
|  | firstorder_10Percentile |  | glszm_GrayLevelNonUniformity |
|  | firstorder_90Percentile |  | glszm_GrayLevelNonUniformityNormalized |
|  | firstorder_Energy |  | glszm_GrayLevelVariance |
|  | firstorder_Entropy |  | glszm_HighGrayLevelZoneEmphasis |
|  | firstorder_InterquartileRange |  | glszm_LargeAreaEmphasis |
|  | firstorder_Kurtosis |  | glszm_LargeAreaHighGrayLevelEmphasis |
|  | firstorder_Maximum |  | glszm_LargeAreaLowGrayLevelEmphasis |
| First-order features (N = 18) | firstorder_MeanAbsoluteDeviation | GLSZM texture features | glszm_LowGrayLevelZoneEmphasis |
|  | firstorder_Mean | (N = 16) | glszm_SizeZoneNonUniformity |
|  | firstorder_Median |  | glszm_SizeZoneNonUniformityNormalized |
|  | firstorder_Minimum |  | glszm_SmallAreaEmphasis |
|  | firstorder_Range |  | glszm_SmallAreaHighGrayLevelEmphasis |
|  | firstorder_RobustMeanAbsoluteDeviation |  | glszm_SmallAreaLowGrayLevelEmphasis |
|  | firstorder_RootMeanSquared |  | glszm_ZoneEntropy |
|  | firstorder_Skewness |  | glszm_ZonePercentage |
|  | firstorder_TotalEnergy |  | glszm_ZoneVariance |
|  | firstorder_Uniformity |  |  |
|  | glrlm_GrayLevelNonUniformity |  | glcm_Autocorrelation |
|  | glrlm_GrayLevelNonUniformityNormalized |  | glcm_ClusterProminence |
|  | glrlm_GrayLevelVariance |  | glcm_ClusterShade |
|  | glrlm_HighGrayLevelRunEmphasis |  | glcm_ClusterTendency |
|  | glrlm_LongRunEmphasis |  | glcm_Contrast |
| GLRLM texture features | glrlm_LongRunHighGrayLevelEmphasis | GLCM texture features | glcm_Correlation |
| (N = 16) | glrlm_LongRunLowGrayLevelEmphasis | (N = 24) | glcm_DifferenceAverage |
|  | glrlm_LowGrayLevelRunEmphasis |  | glcm_DifferenceEntropy |
|  | glrlm_RunEntropy |  | glcm_DifferenceVariance |
|  | glrlm_RunLengthNonUniformity |  | glcm_Id |
|  | glrlm_RunLengthNonUniformityNormalized |  | glcm_Idm |
|  | glrlm_RunPercentage_T2 |  | glcm_Idmn |
|  | glrlm_RunVariance |  | glcm_Idn |
|  | glrlm_ShortRunEmphasis |  | glcm_Imc1 |
|  | glrlm_ShortRunHighGrayLevelEmphasis |  | glcm_Imc2 |
|  | glrlm_ShortRunLowGrayLevelEmphasis |  | glcm_InverseVariance |
|  |  |  | glcm_JointAverage |
|  |  |  | glcm_JointEnergy |
|  |  |  | glcm_JointEntropy |
|  |  |  | glcm_MCC |
|  |  |  | glcm_MaximumProbability |
|  |  |  | glcm_SumAverage |
|  |  |  | glcm_SumEntropy |
|  |  |  | glcm_SumSquares |
|  | gldm_DependenceEntropy |  | ngtdm_Busyness |
|  | gldm_DependenceNonUniformity |  | ngtdm_Coarseness |
| GLDM texture features | gldm_DependenceNonUniformityNormalized | NGTDM texture features | ngtdm_Complexity |
| (N = 14) | gldm_DependenceVariance | (N = 5) | ngtdm_Contrast |
|  | gldm_GrayLevelNonUniformity |  | ngtdm_Strength |
|  | gldm_GrayLevelVariance |  |  |
|  | gldm_HighGrayLevelEmphasis |  |  |
|  | gldm_LargeDependenceEmphasis |  |  |
|  | gldm_LargeDependenceHighGrayLevelEmphasis |  |  |
|  | gldm_LargeDependenceLowGrayLevelEmphasis |  |  |
|  | gldm_LowGrayLevelEmphasis |  |  |
|  | gldm_SmallDependenceEmphasis |  |  |
|  | gldm_SmallDependenceHighGrayLevelEmphasis |  |  |
|  | gldm_SmallDependenceLowGrayLevelEmphasis |  |  |
|  | wavelet-LHL_* (N = 93) |  |  |
|  | wavelet-LHH_* (N = 93) |  |  |
|  | wavelet-LLH_* (N = 93) |  |  |
| Wavelets features | wavelet -HLL_* (N = 93) | Los features (N = 93) | Log-sigma-2.0_* (N = 93) |
| (N = 744) | wavelet-HLH_* (N = 93) |  |  |
|  | wavelet-HHL_* (N = 93) |  |  |
|  | wavelet-HHH_* (N = 93) |  |  |
|  | wavelet -LLL_* (N = 93) |  |  |

Note: GLCM, Gray-level co-occurrence matrices; GLRLM, Gray-level run length matrix; GLSZM, Gray-level size zone matrix; GLDM, Gray-level dependence matrix. *The abbreviated representation of feature types
